# Two Cases of Late-Diagnosed Ovotesticular Disorder of Sex Development

**Published:** 2013-10-22

**Authors:** Eiji Hisamatsu, Yoshikiyo Nakagawa, Yoshifumi Sugita

**Affiliations:** Department of Urology, Kobe Children’s Hospital, Kobe, Japan

**Keywords:** Disorder of sex development, Ovotestis, Hypospadias

## Abstract

Ovotesticular disorder of sex development (ovotesticular DSD) is defined as the presence of testicular and ovarian tissue in the same individual. Both external and internal genitalia of patients with ovotesticular DSD display a spectrum of phenotypes. Most children present with ambiguous genitalia in combination with unilateral or bilateral undescended gonads. We experienced two late-diagnosed children who presented with proximal hypospadias and bilateral scrotal gonads. One should consider the possibility of ovotesticular DSD when managing patients with proximal hypospadias even if both gonads are palpable in the scrotum.

## INTRODUCTION

Ovotesticular disorder of sex development is defined as the presence of both testicular and ovarian tissues in the same individual, which may take the form of one ovary and one testis or, more commonly, one or two ovotestes. Most children present with ambiguous genitalia in combination with unilateral or bilateral undescended gonads.[1, 2] Herein we present our experience of two late-diagnosed children with ovotesticular DSD.

## CASE REPORTS

**Case 1**

A 5-year-old boy was referred with hypospadias cripple. At four years of age, he underwent one-stage repair using the onlay island flap technique for penoscrotal hypospadias at another hospital. Repair was unsuccessful. The meatus was located at the penoscrotal junction at the first visit to our hospital. Although the ventral curvature persisted, no visible scar was noted in the urethral plate. Both gonads were palpated in the scrotum. The gonads were considered as testes on palpation.

Orthoplasty for the persistent ventral curvature and tubularized incised plate urethroplasty for meatal advancement was performed. Although the ventral curvature improved, glans dehiscence occurred. It resulted in a subcoronal meatus. Plan for a two-stage repair using a buccal mucosal graft was declined by the family. During the follow-up, nodules suspicious for ovarian tissues were palpated in the upper pole of both testes. Karyotype analysis revealed 46,XX without SRY gene. The levels of follicle stimulating hormone, luteinizing hormone, testosterone and estradiol were 10.18 mIU/ml, 4.03 mIU/ml, 341 ng/dl and less than 10 pg/ml, respectively. Subsequently, he underwent cystoscopy, laparoscopy, and scrotal exploration for evaluation of DSD.

Cystoscopy showed a 6.5-cm vagina connecting to the posterior urethra. Laparoscopy demonstrated that each vas deferens ran toward its respective side of the internal inguinal ring. Neither uterus nor fallopian tubes were noted. Scrotal exploration revealed a firm nodule resembling an ovary in the upper pole of each testis (Fig. 1). The nodules were removed and testicular biopsies were performed. The remaining testes were placed in the scrotum. Histological analysis revealed the removed nodules were normal ovarian tissues, with the margin containing the testicular tissue. The biopsied specimen was immature testicular tissue containing seminiferous tubules and Leydig cells. Testicular atrophy was not recognized 48 months after the surgery. As a result, karyotype and histological analysis confirmed that he had ovotesticular DSD eight years after the first visit. He has developed normal pubertal maturation.

**Figure:**
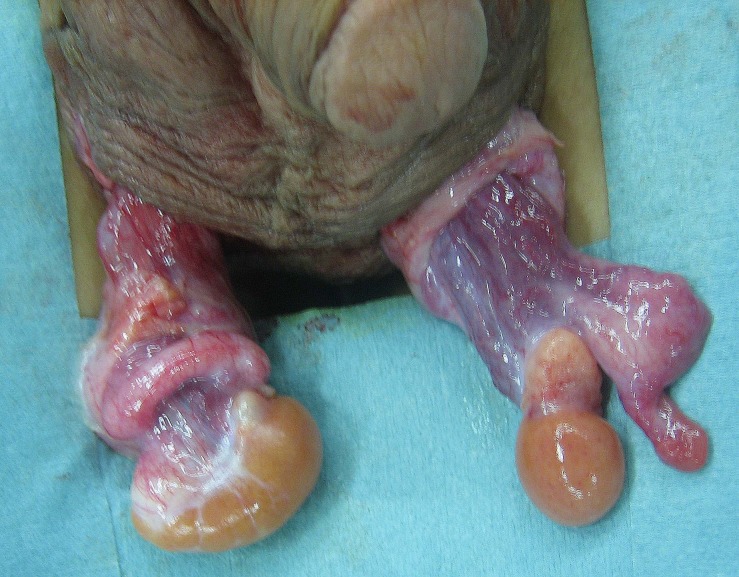
Figure 1: Intraoperative findings of ovotestes. The ovarian portion was firm and yellowish-white in the upper pole, while the testicular portion was soft and brownish-colored in the lower pole.

**Case 2**

A two-month-old boy was referred with proximal shaft hypospadias. On physical examination, penoscrotal transposition was noted in addition to the small phallus. Both gonads were palpated in the scrotum. The gonads were considered as testes on palpation. At two years of age, he underwent one-stage repair using a preputial skin graft without preoperative hormonal stimulation. Glans dehiscence occurred after the surgery, resulting in a subcoronal meatus. At three year of age, he underwent scrotoplasty using a modified Glenn-Anderson technique for penoscrotal transposition. During the follow-up, nodules suspicious for ovarian tissues were palpated in the upper pole of both testes. Karyotype analysis revealed 46,XX without SRY gene. The levels of follicle stimulating hormone, luteinizing hormone, testosterone and estradiol were 0.42 mIU/ml, less than 0.07 mIU/ml, less than 0.05 ng/dl and less than 10 pg/ml, respectively. Subsequently, he underwent cystoscopy, laparoscopy, and scrotal exploration for evaluation of DSD.

Cystoscopy showed a normal urethra without a vagina. Laparoscopy did not clearly demonstrate both vasa deferens. Neither uterus nor fallopian tubes were noted. Scrotal exploration revealed a firm nodule resembling an ovary in the upper pole of each testis (Fig. 2). The nodules were removed and the remaining testes were placed in the scrotum. Histological analysis revealed the removed nodules were normal ovarian tissues, with the margin containing the immature testicular tissue (few germ cells, no Leydig cells). Testicular atrophy was not recognized 19 months after the surgery. As a result, karyotype and histological analysis confirmed that he had ovotesticular DSD. At 10 year of age, the levels of follicle stimulating hormone, luteinizing hormone, testosterone and estradiol were 1.32 mIU/ml, less than 0.07 mIU/ml, less than 0.03 ng/dl and less than 10 pg/ml, respectively. He has not developed pubertal maturation yet.

**Figure:**
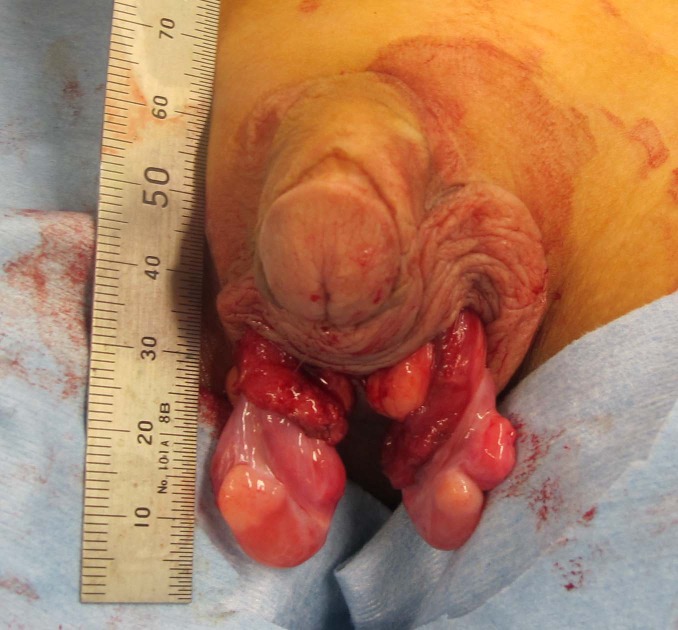
Figure 2: Intraoperative findings of ovotestes. The ovarian portion was firm and yellow in the upper pole, while the testicular portion was soft and pink in the lower pole.

## DISCUSSION

In ovotesticular DSD the ovotestis is the most common gonad, and it may reside at any point along the path of testicular descent.[1,2] Verkauskas et al reported 33 patients with histologically confirmed ovotesticular DSD. The ovotestis was the most frequent gonadal finding (43/66, 65%) in the 33 patients, and 31 of the 43 ovotestes were located in the abdominal or inguinal region.[3] Differentiation of the internal ducts is variable and depends on the function of the ipsilateral gonad. Most children have the unilateral uterus, which may be fully developed. Approximately 60% of children with ovotesticular DSD have a 46,XX karyotype; 33% are mosaics with a second cell line containing a chromosome (46,XX/46,XY; 46,XX/46,XXY), and 7% are 46,XY. [1, 2]

There are few reports of late-diagnosed children with ovotesticular DSD because of unusual presentations.[4-6] Matsui et al reported 8 patients with ovotesticular DSD. One of them presented with perineal hypospadias and bilateral scrotal gonads. The diagnosis of ovotesticular DSD was made 7 years after the first visit. The gonads revealed ovotestes when he underwent orchiopexy for ascending testes to an inguinal region.[6] Our cases also presented with isolated hypospadias with bilateral scrotal gonads.

Karyotyping for all our patients with proximal hypospadias may avoid late diagnosis of ovotesticular DSD. However, routine karyotyping in such cases remains controversial. Although not indicated for isolated anterior and middle hypospadias, the necessity for evaluation of DSD in the setting of concomitant hypospadias and undescended testis (UDT) has been discussed. Kaefer et al evaluated 79 presumed males presenting with UDT and hypospadias. DSD was indentified with nearly equal frequency in the 44 cases (30%) of unilateral UDT and 35 cases (32%) of bilateral UDT. Approximately 15% of patients with hypospadias and a palpable undescended gonad had DSD, while approximately 50% of patients with hypospadias and a unilateral nonpalpable gonad had DSD.[7] On the other hand, McAleer et al do not recommend routine karyotyping in all patients with hypospadias and UDT because the incidence of sex chromosome abnormalities on karyotyping was low (2/48, 4.2%) in their patients.[8]

One should consider the possibility of ovotesticular DSD when managing patients with proximal hypospadias even if both gonads are palpated in the scrotum. In such cases, careful palpation should be performed to rule out asymmetric polar anatomy of the gonads although routine karyotyping is unnecessary.

## Footnotes

**Source of Support:** Nil

**Conflict of Interest:** None declared

